# Convergent evolution of complex structural variants drives therapy resistance in metastatic prostate cancer

**DOI:** 10.1186/s13059-026-04074-2

**Published:** 2026-04-15

**Authors:** Thaidy Moreno-Rodriguez, Meng Zhang, Arian Lundberg, Raunak Shrestha, Martin Sjöström, Anupama Pasam, Joanna Chan, Lisa Devereux, Adam Foye, Xiaolin Zhu, Alana S. Weinstein, Anna S. Trigos, Alexander W. Wyatt, Joshi J. Alumkal, Eric J. Small, Rahul Aggarwal, Scott M. Dehm, Matthew L. Bootsma, Shuang G. Zhao, Mathieu Lupien, Felix Y. Feng, Shahneen Sandhu, David A. Quigley

**Affiliations:** 1 Department of Urology, UCSF, San Francisco, CA USA; 2 Department of Radiation Oncology, UCSF, San Francisco, CA USA; 3 Department of Urology, Emory University, Atlanta, USA; 4 Peter MacCallum Cancer Centre, Melbourne, VIC Australia; 5 Department of Oncology, Sir Peter MacCallum, University of Melbourne, Melbourne, VIC Australia; 6 Division of Hematology and Oncology, Department of Medicine, UCSF, San Francisco, CA USA; 7 Department of Urologic Sciences, Vancouver Prostate Centre, University of British Columbia, Vancouver, BC Canada; 8 Michael Smith Genome Sciences Centre, BC Cancer, Vancouver, BC Canada; 9 Department of Internal Medicine, Division of Hematology and Oncology, University of Michigan Rogel Cancer Center, Ann Arbor, MI USA; 10 Helen Diller Family Comprehensive Cancer Center, UCSF, 1450 3Rd St, Room 387, San Francisco, CA 94158 USA; 11 Masonic Cancer Center, University of Minnesota, Minneapolis, MN USA; 12 Department of Laboratory Medicine and Pathology, Minneapolis, MN USA; 13 Department of Urology, University of Minnesota, Minneapolis, MN USA; 14 Department of Human Oncology, University of Wisconsin, Madison, WI USA; 15 Carbone Cancer Center, University of Wisconsin, Madison, WI USA; 16 William S. Middleton Veterans Hospital, Madison, WI USA; 17 Princess Margaret Cancer Centre, Toronto, ON Canada; 18 Department of Medical Biophysics, University of Toronto, Toronto, ON Canada; 19 Ontario Institute for Cancer Research, Toronto, ON Canada; 20 Department of Epidemiology & Biostatistics, UCSF, San Francisco, CA USA

## Abstract

**Background:**

Targeted therapy prolongs the lives of men with metastatic castration-resistant prostate cancer (mCRPC) but mCRPC is ultimately lethal. DNA copy gains that amplify the Androgen Receptor (AR) gene locus are a key driver of resistance to targeted therapy in mCRPC. Our group has recently shown that extra-chromosomal DNA (ecDNA) frequently drives this amplification. We hypothesized that ecDNA and other complex structural variants (cSVs) also affect other established drivers of therapy resistance in mCRPC and continue to evolve over time. To test this hypothesis, we reconstructed cSV profiles in 193 mCRPC tumors using whole genome and transcriptome sequencing, with matched Hi-C data for 77 tumors.

**Results:**

We identify ecDNA in more than half of mCRPC biopsies and show it frequently amplifies driver genes such as AR and MYC and their non-coding enhancers. The presence of ecDNA is significantly associated with whole genome doubling, chromothripsis, and inactivating TP53 alterations. Deep sequencing analysis of 53 rapid autopsy samples shows cSVs amplifying AR can arise independently within distinct tumors in a single patient. Phylogenetic analysis of tumor evolution implicates this cSV as an early event during metastatic spread. Additionally, a paired analysis of mCRPC samples as patients developed resistance to AR pathway inhibitor (ARPI) therapy demonstrates cSVs evolve in response to ARPI and can be detected in both tumor tissue and circulating tumor DNA.

**Conclusions:**

We conclude that cSVs, particularly ecDNA, are a pervasive contributor to intra-patient heterogeneity in late-stage mCRPC and a key driver of targeted therapy resistance.

**Supplementary Information:**

The online version contains supplementary material available at 10.1186/s13059-026-04074-2.

## Background

One in nine people born with a prostate will develop prostate cancer during their lifetime [1]. Localized prostate cancer is frequently well-controlled with surgery, radiation, or active surveillance. However, metastatic prostate cancer is lethal, with a five year survival rate of approximately 31% [1]. Because prostate tumors depend on androgen signaling to proliferate, systemic androgen deprivation therapy (ADT) is the backbone therapy for aggressive prostate cancer that progresses despite curative intent surgery and/or radiation. Metastatic prostate tumors that develop resistance to ADT are known as metastatic castration-resistant prostate cancer (mCRPC). Androgen receptor pathway inhibitor (ARPI) therapy is the standard of care for men with mCRPC [2, 3], but typically, docetaxel-naïve mCRPC tumors develop resistance to the ARPI enzalutamide in a median of 14 months [4, 5].

The most frequently observed ARPI resistance mechanism is DNA copy gains affecting the androgen receptor (*AR)* gene locus on chromosome X. In approximately 80% of mCRPC tumors, the *AR* and a nearby *AR* enhancer locus gain additional DNA copies through structural variants such as tandem duplications [6, 7]. These gains increase androgen signaling within tumor cells despite castrate levels of testosterone [6–12]. Structural variants can progressively accumulate at the *AR* locus as men receive ARPI therapy [11–13]. Our prior work defining the SV landscape of mCRPC focused on linear SVs, but it is now clear that complex structural variants (cSV) that modify the genome through extrachromosomal DNA (ecDNA) or Breakage-Fusion Bridge (BFB) amplification are a significant contributor to genome evolution in numerous forms of cancer [14–21]. ecDNA, also called double minutes (DM), are large circular double-stranded DNA structures, generally at least one or more megabases in size, that can replicate independently from chromosomes and frequently amplify driver genes [14, 22, 23]. In primary prostate cancer, ecDNA can amplify the oncogene *MYC* [24]*.* The presence of ecDNA amplifying *AR* in mCRPC has been documented previously [25, 26], and we recently showed that ecDNA amplification of *AR* is common in mCRPC [27]. However, the prevalence and heterogeneity of SV and cSV impacting driver genes and their enhancers in mCRPC remain unclear.

Prior studies of mCRPC lesions in a rapid autopsy cohort have reported that most tumors within a patient have similar driver mutation and structural variant profiles [25, 28–30]. However, intra-patient heterogeneity in transcriptional subtypes is increasingly recognized as a significant driver of poor therapy outcomes [31–33]. Obtaining multiple solid tumor mCRPC biopsies from the same individual is rare, which consequently hampers the study of inter-tumor heterogeneity, clonal evolution, and treatment response. We hypothesized that individual mCRPC tumors within a patient would evolve distinct somatic profiles on ARPI therapy administered after metastatic spread, developing alterations that converge on common functional consequences.

To comprehensively assess how cSVs impact resistance to androgen-targeted therapies in patients with mCRPC, we performed deep whole genome, exome, and targeted DNA sequencing and RNA sequencing in 53 castrate-resistant tumors from the CASCADE rapid autopsy cohort. We also evaluated deep whole genome data from the West Coast Dream Team (WCDT) cohort of 140 men, including 77 mCRPC biopsies with matched Hi-C sequencing. To track over time how cSVs evolve in response to ARPI therapy, we evaluated paired pre and post-ARPI tumor biopsies and deep whole genome sequencing from 38 liquid biopsies from men with mCRPC, including four liquid biopsies with matching tumor biopsies.

## Results

### cSVs frequently amplify driver genes in mCRPC

We and others have previously characterized the landscape of simple structural variants in mCRPC [6, 34], such as insertions, tandem duplications, and deletions. In this study, we focused on cSVs that could contribute to resistance to targeted therapy. To assess the frequency and impact of cSVs in mCRPC tumors, we assembled two complementary cohorts that had undergone deep whole genome sequencing at 100 × coverage. The WCDT cohort included 140 mCRPC biopsies that have been described previously [6, 35]. All WCDT tumor samples were obtained from patients undergoing treatment for mCRPC who had progressed on ADT, and in most instances also undergone ARPI therapy at the time of their biopsy. The median age of WCDT patients at the time of their first mCRPC biopsy was 69 years (range 59 to 76). To assess cSV heterogeneity across multiple metastases within an individual patient, we generated new deep whole genome sequencing on mCRPC tumor samples from CASCADE, a research program that enrolls patients with advanced cancer for rapid autopsy after death, and biobanks multiple tumor samples from each donor [36]. The median age of men in CASCADE at the time of their death was 58 years (range 53 to 81). We sequenced 53 tumors from six CASCADE patients who had been treated for mCRPC with multiple lines of therapy, including ADT, radiotherapy, ARPI, taxanes, PARPi, and platinum. All men in CASCADE received at least one course of second-generation ARPI therapy before expiring due to their prostate cancer. Annotation of CASCADE patient therapy history (Additional file 1: Fig. S1a), tumor location, and demographic features are shown in Additional file 2: Table S1.

**Fig. 1:**
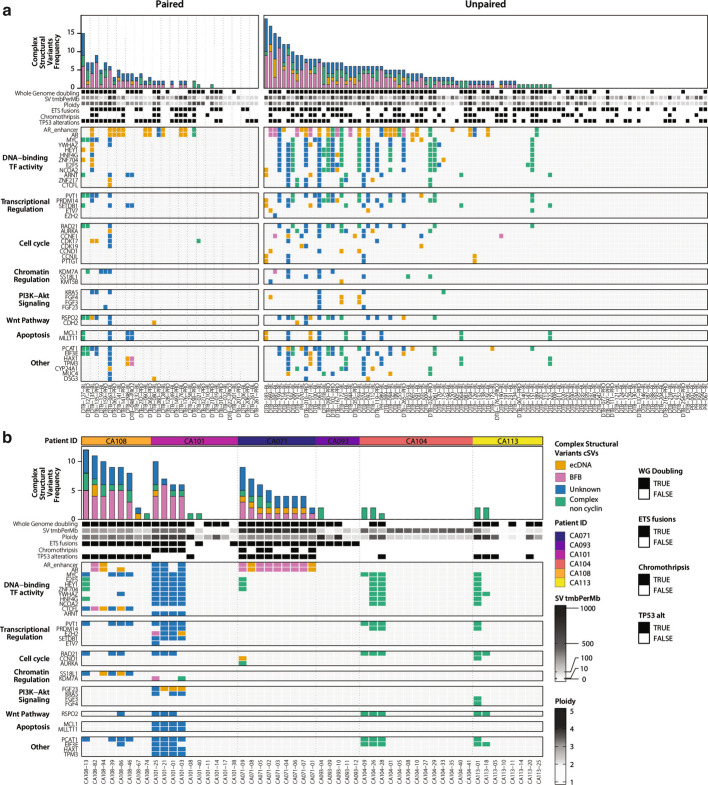
Amplicon distribution in two mCRPC cohorts. Heatmaps showing the frequency and genes impacted in (**a**) WCDT and (**b**) CASCADE patients by four types of complex structural variants: extrachromosomal DNA (ecDNA), Breakage-fusion bridges (BFB), complex-non-cyclical (CNC) and Unknown amplifications when called using AmpliconArchitect

We identified cSVs using AmpliconArchitect [37]. AmpliconArchitect identified four classes of cSVs: ecDNA, Breakage Fusion Bridges (BFB), complex non-cycling (CNC) events, and Unknown events that showed evidence of complex origins but could not be confidently assigned to one of the other classes. Each call reported in this analysis of tissue WGS is listed in Additional file 2: Table S2. A majority of WCDT biopsies had one or more cSVs (63%, 89 of 140 biopsies, Fig. 1a). Most CASCADE autopsy samples (55%, 29 of 53) also had one or more cSVs (Fig. 1b). We concluded that cSVs were frequently present in these mCRPC cohorts.

We then identified the driver genes most frequently impacted by cSVs. As expected, among the driver genes assessed in our study of mCRPC, *AR* was the driver gene most frequently amplified by cSVs. AmpliconArchitect identified ecDNA amplifying *AR* in 16% of WCDT tumors (22 of 140). Furthermore, we identified a long tail of drivers targeted by ecDNA at frequencies of 3% or lower, including *MYC*, cyclin D1 (*CCND1*), *NCOA2*, aurora kinase *(AURKA)*, and fibroblast growth factor 3 *(FGF3),* with the count of events at each driver listed in Table S3*.* Overall, this analysis demonstrated that *AR* and other key drivers of mCRPC are frequently targeted by cSVs, particularly ecDNA.

The presence of ecDNA has previously been linked to tumor genomic instability in tumors [38–40]. To identify events correlated with the presence of ecDNA in our cohorts, we performed an unbiased search for associations between somatic events detectable by WGS and the presence of ecDNA. Tumors harboring ecDNA in WCDT were significantly more likely to harbor increased structural variations overall, to have undergone chromothripsis on the chromosome harboring ecDNA, and to harbor inactivating *TP53* mutations (Additional file 1: Fig. S1b). We validated these observations in the CASCADE dataset (Fig. S1c). We tested whether chromothripsis was more likely to occur on the same chromosome that harbored ecDNA or BFB events and observed a significant association between chromothripsis and ecDNA or BFB at the chromosome (CASCADE: P = 1.6 × 10^–5^ and WCDT: P = 1.7 × 10^–10^, Fisher’s Exact Test). We and others have previously linked *TP53* mutations to chromothripsis in mCRPC^6^ and other malignancies [16, 38, 40–42]. These results were consistent with the fact that ecDNA formation requires double-strand breaks and tumors with greater genomic instability present more opportunities to develop cSVs including ecDNA. From these analyses, we concluded that cSVs are common in mCRPC, and most frequently amplify *AR*. In some cases, two or more oncogenes can be amplified in a single event (Additional file 1: Fig. S1d, e). In summary, cSVs impact key drivers of mCRPC and were most frequently detected in tumors with *TP53* inactivation.

### Chromatin contact maps provide orthogonal support for cSVs

**Fig. 2:**
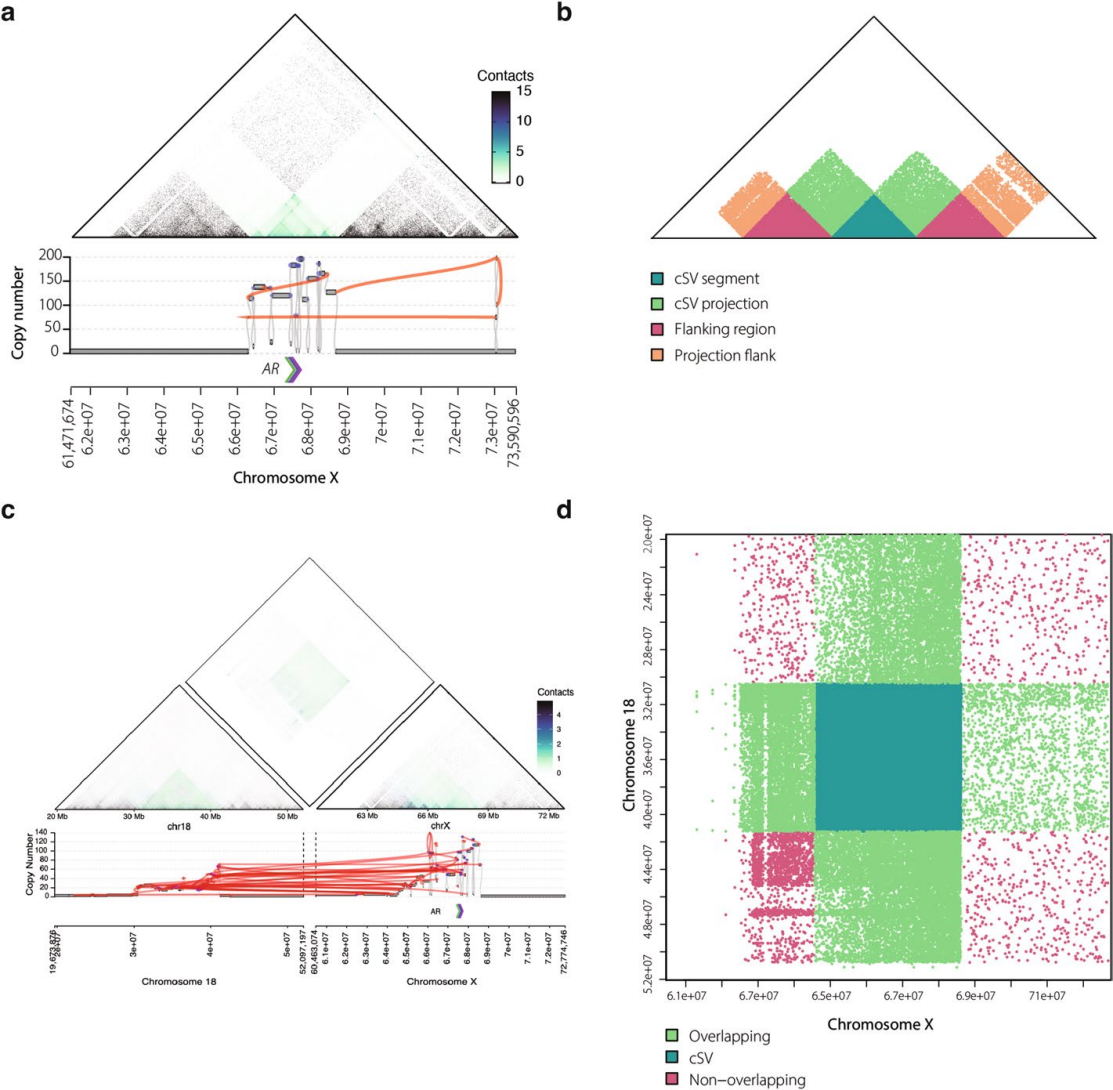
Hi-C provides evidence of ecDNA and other cSVs in mCRPC. **a** At the top, a Hi-C 10 Kb. contact map of a mCRPC tumor at the *AR* locus on chromosome X, where darker points indicate a stronger DNA-DNA contact signal. Below, the reconstructed copy number segments, connected using genome graphs of an ecDNA amplicon. **b** An illustration of the regions in panel (a) labeled to statistically evaluate cSV from Hi-C data. Region bounds are defined by AmpliconArchitect/AmpliconClassifier analysis, see Methods. **c** Hi-C inter-chromosomal contact map of an ecDNA predicted to include segments from two chromosomes (chr18, left; chrX, right; chr18-chrX, top-middle). Below are the reconstructed copy number segments in both chromosomes, connected using the genome graphics of an ecDNA amplicon. **d** An illustration of the regions in panel (c) labeled to illustrate the testing space for statistical analysis in the presence of cSVs

Hi-C sequencing is an unbiased whole-genome method that quantifies the contact frequency between any two genomic regions [43, 44]. We have previously demonstrated that Hi-C sequencing of mCRPC biopsies can detect events consistent with ecDNA amplification of the *AR* locus by identifying regions with unexpectedly low contact to adjacent un-amplified DNA [27]. In this study, we evaluated whether Hi-C could serve as orthogonal support for all cSVs calls identified in the WCDT cohort. We used 48 WCDT samples where both whole genome sequencing and Hi-C assays had been performed and at least one cSV event was nominated by AmpliconArchitect analysis (Fig. 1a). As a proof of concept, we examined a WCDT biopsy where AmpliconArchitect predictions indicated an ecDNA-driven amplification at the *AR* locus and then visually inspected the matching Hi-C data. The contact maps showed a clearly defined region of low contact within segments overlapping the amplified region compared to the surrounding segments, matching the breakpoints identified by WGS (Fig. 2a). This demonstrated that ecDNA coincided with defined boundaries of low versus high contact in Hi-C data. To statistically identify these events, we defined specific regions: the amplified event region and flanking regions, which each have high internal contact, and the adjacent projection space where low inter-region contact may be linked to a cSV (Fig. 2b). Statistical results are summarized in Additional file 2: Table S4, with details provided in Methods. To extend this approach to cSVs that span multiple chromosomes, we analyzed a WCDT biopsy with ecDNA-driven amplification where the event included both chromosomes X and 18. The Hi-C contact maps showed a region of high interaction within the amplified segments of both chromosomes (Fig. 2c), illustrating a characteristic Hi-C signature of ecDNA with trans-interactions. Analogous to the single-chromosome model, we defined event and overlapping regions (Fig. 2d) to identify regions with statistically significant contact between chromosomes. Multi-chromosome statistical results are summarized in Additional file 2: Tables S5 and S6.

We then systematically evaluated all cSV calls from AmpliconArchitect to test for Hi-C contact patterns consistent with the presence of a cSV. Statistical significance was defined by comparing summarized contact between amplified and adjacent un-amplified regions of DNA (see Methods). This approach was applied to 2,137 DNA segments across 219 cSVs, many of which involved multiple discontinuous segments stitched together into a single amplified region (as illustrated in Fig. 2a). In total, we assessed 219 candidate cSVs that comprised 1,074 segments identified by WGS. Of the 219 candidate cSVs, 182 were cis-interactions confined to a single chromosome. The Hi-C analysis supported 68% of AmpliconArchitect candidate cSVs (124 of 182), defined by the presence of at least one significant segment when comparing event projection to flank projection. All cis-interacting Breakage Fusion Bridge (BFB) events (8 of 8) and ecDNA (11 of 11) involving the *AR* locus were supported. The remaining 37 events were trans-acting, of which Hi-C supported 54% of events (20 of 37). All trans-interacting ecDNAs (2 of 2) involving the *AR* locus were also supported. Examples of predicted ecDNA with and without Hi-C support are shown in Additional file 1: Fig. S2. We hypothesized that events in samples with higher tumor purity and events with higher total copy number would be more likely to be supported, as both factors could increase the signal-to-noise ratio for cSV detection. Consistent with this, we observed significant associations of evidence with both tumor purity (*P* < 2.2 × 10^–16^, Kruskal–Wallis test) and event-weighted copy number (*P* < 4 × 10^–12^, Kruskal–Wallis test). BFB cycles can produce contact patterns called neo-loops that can be distinguished from those produced by ecDNA using Hi-C analysis [21]. We identified neo-loops present within BFB events at the predicted junctions in a subset of samples, when compared to non-amplified samples in the same region (Additional file 1: Fig. S3a-d). However, we failed to detect neo-loops using the same criteria in other samples with equally strong evidence for BFB events from AmpliconArchitect. This may reflect the impact of tumor purity, copy-number heterogeneity, and sequencing depth on the magnitude of these signals. Taken together, these analyses demonstrate that computationally predicted ecDNA and BFB events were well-supported by the orthogonal Hi-C assay.

### cSVs frequently target non-coding regulators of mCRPC drivers

ecDNA frequently amplifies regulatory DNA as well as driver genes [40, 45–49]. We observed that 70% of the ecDNAs and 42% of BFBs amplified putative enhancer regions without any known driver genes (134 out of 193 ecDNAs and 11 out 26 BFBs, respectively). We have previously shown that the *AR* enhancer, located 624 Kb upstream of the *AR* gene body, is more frequently amplified than the *AR* gene body [6]. This led us to hypothesize that cSVs would frequently target this and other enhancer regions. Indeed, 64% (123 of 193) of the cSVs classified by AmpliconArchitect as ecDNA in the WCDT cohort included a super-enhancer region (Additional file 2: Table S7). Then, we used public chromatin immunoprecipitation followed by sequencing (ChIP-seq) data for H3K27ac, H3K4me2, and H3K4me3 histone marks in prostate tumors, and H3K27ac marks in PDX models [50], to interrogate whether these were prostate lineage-specific regulatory elements. We found that 78% and 73% of ecDNAs (151 out of 193 and 141 out of 193, respectively) overlapped with H3K27ac peaks (Additional file 2: Table S8). To evaluate whether the observed overlap between ecDNAs or BFBs and H3K27ac peaks was greater than expected by chance, we performed a resampling test, randomizing genomic intervals while preserving their size and chromosome distribution, and masking unmappable regions drawn from the ENCODE Project ENCFF356LFX exclusion list. Regions defined by ecDNAs were significantly enriched for H3K27ac peaks (*P* = 0.02), whereas there was not a significantly enriched overlap between regions defined by BFBs and H3K27ac sites (*P* = 0.134). We exclusively observed focal amplification of enhancers associated with AR and MYC, absent amplification of the corresponding gene body, as a product of linear or ecDNA-driven DNA copy gain as opposed to BFB-associated copy gain. Additionally, when comparing them with a dataset of super-enhancers [51], 56% of the ecDNAs lacking oncogenes overlapped with a known super-enhancer (75 out 134) (Additional file 2: Table S9). In summary, ecDNAs, but not BFBs, preferentially co-localized with regions marked by active enhancers, supporting the biological relevance of the observed overlap.

**Fig. 3:**
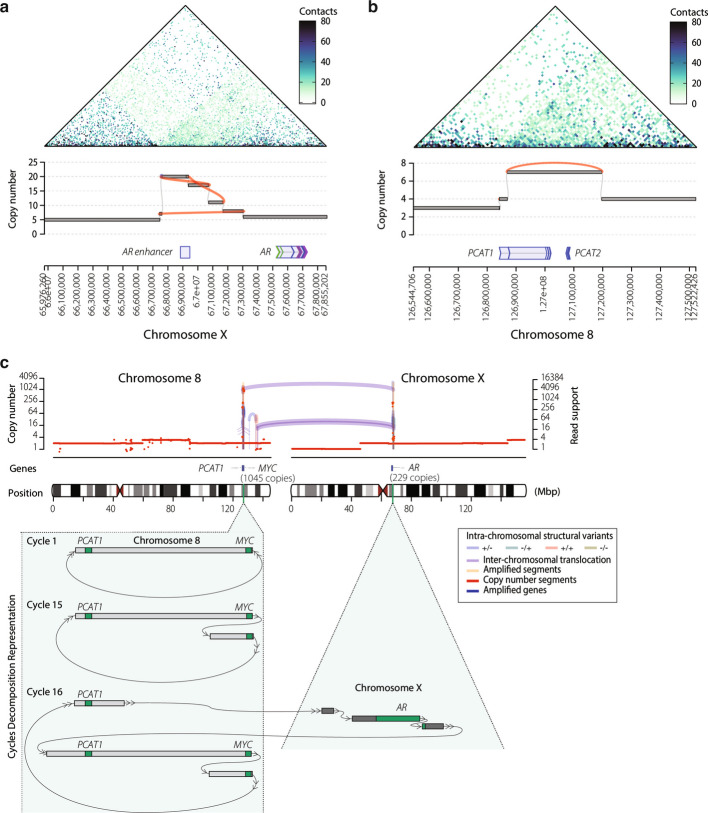
Enhancers and other non-coding regulatory elements are amplified by ecDNA in mCRPC. **a** Combined Hi-C and copy number graph plots show an ecDNA that amplified only the *AR* enhancer but not the *AR* gene body. **b** Combined H-C and copy number graph plot shows an ecDNA that amplified the *PCAT1* locus. **c** ecDNA amplicon that co-amplified *AR* and *MYC*. Cycles identified are represented below. Scale for DNA copy number and read support level are shown on left and right axes, respectively. Arcs connecting genomic segments link amplicon breakpoints, with colors indicating read orientation

Our unbiased, genome-wide analysis revealed that the *AR* enhancer was the most commonly amplified genomic region by ecDNA and cSVs overall (Fig. 3a, Additional file 1: Fig. S4a). This enhancer site was amplified by cSVs in 29% of WCDT cases (41 of 140 biopsies), most frequently by ecDNA (17%, 24 of 140 biopsies); three of those cases amplified the *AR* enhancer only (Fig. 3a), and BFB events (5%, 7 of 140 biopsies). This prompted us to investigate further for additional regulatory regions amplified by cSVs. We found additional frequent cSVs in two regions on the long arm of chromosome 8. The first region was located within the 8q24.21 gene desert and amplifies prostate-associated [52] long non-coding RNAs including *PRNCR1*, *PCAT1*, and *PCAT2*, located approximately 750 Kb upstream of oncogenes *MYC* and *PTV1* (Additional file 1: Fig. S4b). Notably, *PCAT1* functions as a prostate cancer-specific super-enhancer, while *PVT1* recruits EZH2 to promote androgen-independent prostate cancer by suppressing the androgen receptor [53]. We observed cSVs amplifying *MYC* alone, *PCAT1* + *MYC* (one case), *MYC* + *PVT1* (one case), and *PCAT1* + *PCAT2* without *MYC* (Fig. 3b) (three cases). In one unusual case, both *AR*, *PCAT1*, and *MYC* were co-amplified in the same ecDNA amplicon (Fig. 3c). The second frequently amplified regulatory region on chromosome 8 spans from 8q21.11 to 8q21.13 and is marked by H3K27ac. This region also includes coding genes involved in transcriptional regulation, such as *HNF4G*, *HEY1*, *E2F5*, *ZNF704*, and *SNX16* (Additional file 1: Fig. S4b). We concluded that non-coding enhancer regions are among the most frequent cSVs targets in mCRPC. To assess whether copy number gain via ecDNA correlated with increased expression for these genes, we fit a linear model to assess whether ecDNA status was associated independently with transcriptional levels when added to a model including copy number (CN) status. After correction for multiple testing, in the WCDT cohort we found significantly improved model fit for *AR* (F = 11.36, *p* = 2.58 × 10⁻5, FDR = 1.03 × ^10^⁻4), *PCAT1* (F = 3.82, *p* = 0.024, FDR = 0.032), and *CCND1* (F = 6.77, *p* = 1.54 × 10⁻3, FDR = 3.09 × ^10^⁻3). These results were consistent with the model that ecDNA amplification contributed to gene transcription beyond copy number alone.

### Functionally convergent cSVs arise in distinct tumors within patients

**Fig. 4:**
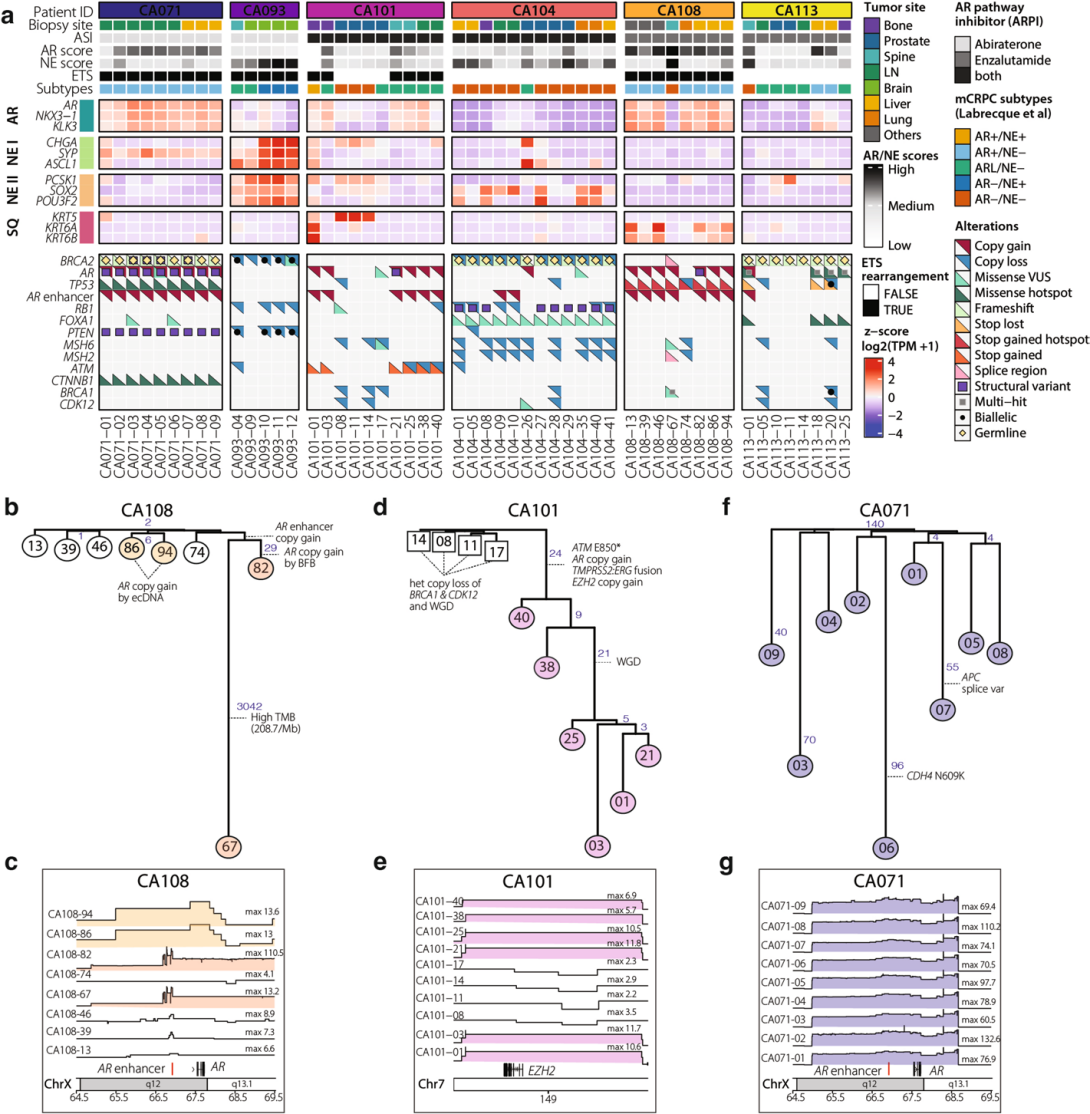
Convergent evolution of ecDNA in patients with end-stage mCRPC. **a** Heatmap showing expression levels of key androgen signaling and neuroendocrine gene markers in 52 CASCADE tumors, expressed as the z-score of log2(TPM + 1). Somatic alterations are represented according to the color legend, including hotspot mutations and variants of unknown significance (VUS). Multiple independent hotspot mutations are annotated with the number of hits. **b**,** d**,** f** Phylogenetic trees from patients CA108, CA101, and CA071, constructed using Neighbor-joining (NJ). Node distances, indicated as numbers on branches, show the pairwise distances between nodes calculated using the number of deleterious somatic mutations shared by all nodes under the line. Tumors are leaf nodes with squares for tumors obtained from the prostate site at the time of autopsy and circles for distant metastasis. Alterations noted with a dashed line are shared by all nodes under the line. **c**,** e**,** g** Copy number profiles from CA108, CA101, and CA071 of the *AR* or *EZH2* loci in all tumors from these CASCADE patients. The maximum copy number for each tumor is noted on the right side of each copy profile. TMB: tumor mutational burden; WGD: whole genome doubling; splice var: splice variation; SV:del: inactivating structural variation producing a deletion; HR: homologous recombination; het copy loss: heterozygous copy loss. Colors in plots (**c**,** e**,** g**) highlight tumors with similar copy profiles

We next used the CASCADE autopsy cohort to evaluate functional convergence among cSV that might arise independently across different tumor sites within an individual. To trace the evolutionary relationships between spatially distinct autopsy samples within men with mCRPC, we performed phylogenetic reconstruction using pathogenic mutations identified in deep exome (300X coverage) and targeted DNA sequencing (5000X coverage) of the tumors. Our analysis revealed that cSVs amplifying *AR* arose multiple times independently in patient CA108. All CA108 tumors harbored the same pathogenic *TP53* mutation, indicating their common origin (Fig. 4a). On one evolutionary branch, tumors CA108-86 and CA108-94 both harbored ecDNA amplifying *AR* with similar breakpoints (Fig. 4b, 4c, Additional file 1: Fig. S5a). On another branch, tumor CA108-82 harbored a distinct BFB with a focal tandem duplication at the *AR* enhancer amplification of more than 100 copies (Fig. 4c). Three other tumors from this patient (CA108-46, 39, and 13) all harbored distinct focal *AR* enhancer linear amplifications.

We sequenced four distinct areas from the primary prostate tumor and six metastatic tumor samples from patient CA101. All metastatic tumors contained a *TMPRSS2-ERG* fusion and *ATM* gene inactivation that was absent in the prostate-derived tumor samples. As *TMPRSS2-ERG* is a clonal, tumor-initiating alteration, this indicated the metastatic samples we sequenced were derived from a precursor clone that was not sampled in this analysis (Fig. 4d). All CA101 metastatic tumors harbored *AR* enhancer amplification that was absent in the prostate tumor regions sampled. In comparison to the prostate-derived CA101 samples, all metastatic CA101 samples amplified Enhancer of Zesty Homolog 2 (*EZH2*), a transcriptional repressor frequently amplified in mCRPC [54]. AmpliconArchitect reported that samples CA101-38 and CA101-40 amplified *EZH2* by linear amplification, while samples CA101-01, 03, 21, and 25 amplified *EZH2* via cSVs (Fig. 4e). However, all six regions shared overlapping breakpoints and had high Jaccard similarity scores (Additional file 2: Table S10, Additional file 1: Fig. S5b). This supports an interpretation that amplification in all six samples resulted from a common event, but lower tumor purity levels in samples CA101-38 and CA101-40 precluded AmpliconArchitect from assigning the same call to all six samples.

In contrast to the evolutionary diversity observed in patients CA108 and CA101, all nine tumors from patient CA071 harbored a region of high-level *AR* and *AR* enhancer amplification by either ecDNA or BFB with identical structural variant breakpoints, consistent with cSVs developing during ADT exposure prior to dissemination (Fig. 4f, g, Additional file 1: Fig. S6). All nine CA071 tumors also shared identical alterations in *PTEN, TP53,* and *CTNNB1,* consistent with a common cell of origin (Fig. 4a). All CA071 tumors were classified *AR*-high/NE-negative by transcriptional subtyping (Fig. 4a) [32, 35]. Tumors CA071-03 and CA071-06, both located at para-aortic lymph nodes, additionally harbored an identical sub-clonal *FOXA1* p.Asp249Asn alteration affecting the *FOXA1* wing-2 domain, with variant allele frequencies of 26% and 2%, respectively. FOXA1 is a key AR pioneer factor, and wing-2 domain mutations are frequently observed in primary prostate tumors, where they have been described as mutually exclusive with ETS fusion rearrangements [55, 56]. The emergence of a *FOXA1* mutation in two of nine deeply sequenced metastatic lesions, all of which harbored an identical ETS fusion (Fig. 1b), is consistent with a model in which *FOXA1* wing-2 mutations may be mutually exclusive with ETS fusions in the localized setting, but can be observed in metastatic tumors harboring ETS fusions.

### cSV evolution tracked during tumor progression

**Fig. 5:**
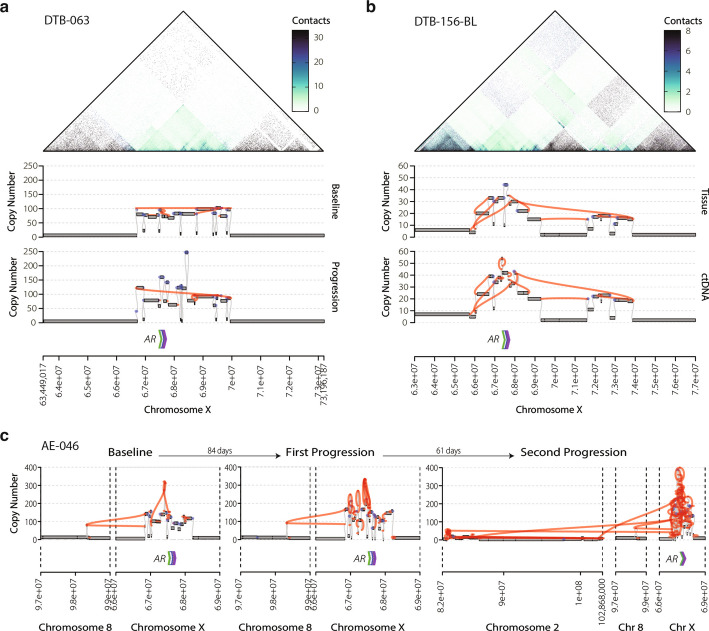
Tracing cSV evolution over time using serial solid and liquid biopsies. Reconstruction of *AR* ecDNA across multiple tissue samples from a single patient. **a** From top to bottom, Hi-C 10 Kb contact map from baseline tissue at the *AR* locus, matched copy number segments, and reconstructed complex structural variant genome graphs of an ecDNA from the same sample at baseline (above) and progression on ARPI (below). **b** From top to bottom, Hi-C 10 Kb contact maps from a tumor biopsy at the *AR* locus, genome graph and junctions from a tumor biopsy, and genome graph and junctions from matched ctDNA sample from the same patient. **c** Genome graphs tracking the evolution of AR cSVs detected in plasma samples from a single patient after progression on two ARPI therapies

To directly assess how cSVs evolve when mCRPC tumors are exposed to ARPI therapy, we next interrogated serially collected tumor samples obtained from mCRPC patients receiving ARPI therapy. We first analyzed 40 recently published mCRPC paired tumor biopsies. Baseline samples were obtained after progression on ADT but prior to commencing ARPI therapy, and progression samples were obtained after progression on ARPI [12]. We identified cSVs affecting the *AR* locus in eleven tumors. Eight cSVs were detected in both paired baseline and progression tumor samples (Fig. 1a, Fig. 5a). Calculating the Jaccard similarity score supported the model that these cSVs originated from a common event (Additional file 2: Table S11). Two other cSVs were present only in the baseline biopsies, and one cSV was only present in the progression sample; however, in this patient, the solid biopsies were taken from different locations, in line with our observation from the rapid autopsy patients. Samples taken at progression on ARPI had additional structural variants affecting the *AR* locus (Fig. 5a), providing further evidence that cSV are maintained under selective pressure and continue to evolve throughout therapy as observed in previous studies [11]; Additional file 1: Fig. S7 illustrates an additional example of this phenomenon.

Obtaining serial biopsies of tumors from the same patient is generally challenging, and it is rarely feasible. Therefore, we assessed whether cSVs could be detected in circulating tumor DNA (ctDNA). We interrogated 41 plasma samples obtained from 22 patients using previously published deep WGS of plasma from patients with mCRPC [11]. Fourteen of the 22 patients had serial timepoints, ctDNA collected at baseline, and after progression on an ARPI therapy. To validate the use of liquid biopsies for cSVs detection, we directly compared three cases where deep WGS of a tumor and ctDNA samples were available from the same patient at the same timepoint. Calculating the Jaccard similarity score, we evaluated whether tissue and ctDNA identified similar cSVs structures. We found similar amplification boundaries with ecDNAs or other cSVs predicted in both cases (Fig. 5b, Additional file 1: Fig. S8a-b, Additional file 2: Table S12). We identified cSVs in 38 of the 41 ctDNA samples (Additional file 2: Table S13). *AR* and the *AR* enhancer were co-amplified by cSVs in 16 of 38 cases. Similar to our analysis of tumor tissue, four of 14 patients with matched liquid biopsies samples exhibited consistent cSVs amplifying *AR* at both timepoints (Additional file 2: Table S14). Additionally, we identified two cases where cSVs were only detectable after progression on ARPI. In one ctDNA case with two progression samples of plasma taken at distinct timepoints after progression on ARPI therapies, we observed the continued evolution of cSVs affecting AR (Fig. 5c). Our analysis of multiple tumor samples in CASCADE suggests that this may be indicative of evolution at distinct tumor sites.

## Discussion

Despite the well-established survival benefits from ARPI therapy for men with advanced prostate cancer, treatment resistance ultimately limits its effectiveness. Most castrate resistant prostate tumors continue to grow in response to androgen signaling, and amplifications of *AR* and other driver genes are typically observed in advanced disease. We have shown that ecDNA and other cSVs can be identified in mCRPC tumors from both patients who had exhausted all treatments (as seen from the CASCADE samples) and in patients still receiving ARPI or other forms of systemic therapy (WCDT cohort). These cSVs amplify non-coding regulators and key oncogenic drivers in prostate cancer, including *AR*, cyclins, and *MYC*. The amplification of *AR* and a nearby *AR* enhancer region by ecDNA was observed in 16% of mCRPC tumors. The recurrent targeting of *AR* and its enhancer by cSVs underscores the critical role of AR signaling in mCRPC, even against the backdrop of advanced therapies. Moreover, ecDNA or BFB can occur repeatedly within the same patient. Our observations in rapid autopsy patients highlight convergent evolution across physically distinct tumors to amplify *AR* and its enhancer by both simple DNA copy gain and more complex cSVs. Recent studies have demonstrated the importance of ecDNA in tumor heterogeneity and therapy resistance [14, 17, 18, 23, 57]. Analysis of matched tumor sampling taken prior to and after the progression of ARPI therapy demonstrated that cSVs continue to evolve at the *AR* locus during ARPI treatment. Given the association previously shown in retrospective data between the presence of ecDNA amplifying *AR* and worse outcomes for men with mCRPC receiving ARPI therapy [27], we speculate as tumors accumulate increasing amounts of ecDNA amplifying *AR* over time this confers a selective advantage over tumors with linear amplifications alone.

DNA damage producing structural alterations is a prerequisite for the formation of cSVs. We showed ecDNA is more frequently present in genomes of mCRPC with chromothripsis and *TP53* mutations. Although our data cannot establish a causal relationship, we observe that ecDNA was commonly located on the chromosome that underwent chromothripsis, which suggests a possible link. Other studies have demonstrated a correlation between ecDNA frequency and exposure to DNA-damaging agents such as taxanes [19] and methotrexate [58]. For the first time, we profiled cSVs and, specifically, ecDNA in liquid tumor biopsies from mCRPC patients. Our findings demonstrate that WGS of ctDNA can detect and track the evolution of ecDNA over time. Our data are consistent with prior observations [11, 12] that when AR-positive prostate tumors with *AR* copy gains are exposed to ARPI, they tend to accumulate additional copy gains to *AR* or a nearby *AR* enhancer and retain their AR-positive lineage. These findings do not preclude the possibility that small AR-negative subclones exist within these tumors and that such subclones could develop novel cSVs after ARPI exposure that provide a competitive advantage, changing the overall phenotype of the tumor. Future studies employing serial biopsies with single-cell or spatial resolution would help to clarify this question. Prior studies have also identified microDNA, another type of circular DNA fragment, in liquid biopsies. These structures, which are less than ~ 50 kb, can be released by both normal and tumor cells (Reviewed in [59]). These smaller circular DNA fragments have been observed in plasma samples from patients harboring lung and ovarian cancer tumors [60] and in maternal plasma, where the source is fetal tissue [61]. MicroDNA has also been detected in urine sediments from patients with urothelial bladder carcinoma [62]. More recently, Knutson et al. developed *AR*-ctDETECT, an assay to detect alterations in *AR* and other oncogenes in plasma from mCRPC patients [63]. This study identified amplification of *AR* and other driver genes using plasma DNA samples and inferred that high-level amplification was likely due to the presence of cSVs. Whole genome sequencing or other specialized methods may provide more direct evidence for the presence of a cSV. Collectively, these studies show that it is possible to detect and monitor the evolution of ecDNA using non-invasive biopsies.

Identifying cSVs and distinguishing between types of cSVs using short-read sequencing is a complex challenge. The landscape of complex structural variation includes chromothripsis [64], chromoplexy [65], and other events that do not always produce extreme copy gain. In this report, we focused on ecDNA and BFB events linked to copy gain because driver gene copy gain is often linked directly to therapy resistance. Although long-read sequencing or optical genome mapping methods may be more definitive for cSV isoform reconstruction, these methods generally require high molecular weight DNA to be maximally effective, which is not always possible with small tumor biopsies. We demonstrated that analysis of matched samples using both WGS and Hi-C sequencing allowed orthogonal, unbiased support for many computationally identified cSV events. While Hi-C analysis revealed neo-loops at BFB-predicted junctions in a subset of samples, we were unable to reliably distinguish between ecDNA and BFB events using neo-loop analysis based on Hi-C contact patterns alone across our cohort. This limitation highlights the need for orthogonal approaches or improved computational methods to resolve cSV identity in heterogeneous clinical samples. We observed cases where alterations with the same bounds were identified as either ecDNA or BFB events in physically distinct tumors from the same individual. This may partly reflect biological reality in that BFB events can produce ecDNA [16, 66], and ecDNA can be reincorporated back into genomic chromosomes producing HSR events [57, 67].

Our primary tool for cSV analysis was AmpliconArchitect/AmpliconClassifier, supplemented with analysis of matched Hi-C data when possible. In many cases, this analysis unambiguously supported a single cSV interpretation. However, computational reconstruction of cSV events from bulk WGS is not definitive, and the calls produced by our pipeline were also influenced by technical artifacts and stochastic variation. We identified highly amplified DNA copy number and higher tumor purity as features correlated with agreement between WGS and Hi-C analysis of cSV calls. In some cases, ecDNA can emerge from a BFB site during the genomically unstable process of BFB cycles [66]. We cannot directly observe this process with our data, but this may explain some cases where we report both BFB and ecDNA events in two or more samples with a common cell of origin and partially overlapping SV breakpoints. These divergent cases may also be influenced by small variations in observed data near the heuristic thresholds that influence the output of the AmpliconClassifier pipeline. We provide output of AmpliconArchitect used for each call in our study to aid in the interpretation of specific instances (Additional file 2: Table S15). In genomes where multiple variations have accumulated, including variations present only in sub-clonal cell populations, studies using bulk sequencing alone may be insufficient to deconvolute the evolutionary history of the tumor. Future studies using single cell analysis methods may be required to accurately reconstruct the evolutionary history of tumor cells.

## Conclusions

Here, we reconstructed cSV profiles in 193 mCRPC tumors using whole genome and transcriptome sequencing, with matched Hi-C data for 77 tumors. Our analysis integrating deep whole-genome sequencing with Hi-C data provides robust support for variant identification. We show that complex structural variants, particularly extrachromosomal DNA and breakage–fusion–bridge amplifications, are pervasive features of mCRPC. These variants most frequently amplify the androgen receptor locus and its distal enhancer, but they also recurrently target other oncogenic drivers and lineage-specific non-coding regulatory elements including super-enhancers. Complex structural variants most often arise in genomically unstable tumors characterized by *TP53* inactivation and chromothripsis. By studying multiple metastatic sites within individual patients, we identified both shared and independently acquired cSVs, supporting a model of convergent evolution amplifying AR signaling in AR-dependent mCRPC. Longitudinal analyses of paired tumor biopsies and circulating tumor DNA demonstrate that cSVs are maintained under therapeutic pressure and continue to evolve during treatment. The ability to detect and track ecDNA and related cSVs in liquid biopsies highlights their potential as non-invasive biomarkers of tumor evolution and therapy resistance. Collectively, these results support a model in which repeated amplification of *AR* and associated regulatory elements via cSVs provides a selective advantage during ARPI therapy.

## Methods

### CASCADE and WCDT sample processing

Recruitment to the CASCADE (Cancer tiSsue aCquisition After DEath) rapid autopsy program at the Peter MacCallum Cancer Centre followed approved protocols and the rapid autopsies were performed as previously described [36].Patients with mCRPC who had largely exhausted most conventional treatments, including ADT, ARPI, chemotherapy, and, if relevant, a PARPi, were enrolled in the cohort. The clinical characteristics of CASCADE patients in this study are available in Additional file 2: Table S1. Tissue collections were undertaken within a median of 7.7 h (range: 4–14). Fresh-frozen metastatic samples were individually macro-dissected in individual petri dishes to avoid cross-contamination and flash frozen. H&E sections of the tumor blocks were obtained and confirmed by the pathologist. DNA from frozen sections was extracted with the QIAamp Fast DNA Tissue Kit (Cat#51,404) from QIAGEN. Germline DNA was extracted with the QIAamp DNA Blood Mini Kit (Cat#51,106) from QIAGEN.

RNA was extracted with the RNeasy UCP Micro Kit (Cat#73,934) from QIAGEN. Barcode-indexed sequencing libraries were generated with Kapa Hyper Prep reagents (Kapa Biosystems-Roche, Basel, Switzerland) from genomic DNA samples sheared on Covaris and Bioruptor Pico instruments. Samples were indexed using Unique Dual Indexed Y-Adapters. One PCR cycle was performed, and whole genome libraries were sequenced on NovaSeq 6000 S4 150PE flowcells. Exome capture was done on the library pools using the IDT xGen hybridization capture kit (Integrated DNA Technologies, Coralville, IA) using the xGen Exome Research Panel v1.0. Targeted capture was performed using a custom IDT Discovery Panel targeting 116 genes and processed using the same hybridization method as the xGen Exome panel. Image-guided fresh-frozen frozen metastatic castration-resistant tissue biopsy samples were collected, and DNA was extracted as previously described [6].

### WGS data processing

Quality controls were implemented using FastQC https://github.com/s-andrews/FastQC version 0.11.8 on the raw sequence data to assess yield and raw base qualities. Qualimap [68] version 2.2.1 was used to determine coverage, mapping qualities, and bias for GC-rich bins. Reads were aligned using bwa mem version 0.7.17-r1198-dirty [69] against reference genome NCBI GRCh38 PAR-masked with decoys hs38d1. Subsequent analytical steps used also this reference genome unless otherwise specified. Read duplicates were marked for filtering using Picard version 2.23.8 MarkDuplicates http://broadinstitute.github.io/picard/. Bam files were sorted and then indexed using samtools version 1.9–93-g0ca96a4 [70]. Sample relatedness was determined using SMaSH https://github.com/rbundschuh/SMaSH across the tumors from the same patient.

### Variant calling and filtering

Structural variants were determined using GRIDSS [71] version 2.12.2 in a joint call on all samples from the same patient. Output was filtered, split to individual tumors, and post-processed using GRIPSS version 1.11. Somatic mutations analysis was performed with MuTect2 [72] version 3.1.0 and Strelka2 [73] version 2.9.10 using Manta [74] version 1.6.0–3-g75b5c38 indels as input. High confident list of somatic mutations list was generated using the intersection of Strelka2 and MuTect2.

Germline mutation analysis was performed following the best practices workflow for germline variants GATK HaplotypeCaller version 4.2.2.0, annotated using SnpEff and SnpSift [75, 76] version 4.3t (build 2017–11–24 10:18). Variants of interest were identified as those labeled as “pathogenic” or “likely_pathogenic” and “missense”, “stop_gain” or “frameshift” on the “CLNSIG” field.

### Determining copy number status

Copy number variants were identified using the hmftools suite (https://github.com/hartwigmedical/hmftools). Briefly, read depth ratios and GC normalization were performed by COBALT version 1.11, the B-allele frequency (BAF) was calculated by AMBER version 3.5, and copy number alterations and tumor purity and ploidy were determined using PURPLE version 3.0 by combining the output from GRIDSS, Strelka, COBALT, and AMBER. Fusions were predicted using LINX [77] version 1.17. Calls were manually reviewed using the Integrated Genome Viewer [78] version 2.9.4.

Gene copy number was calculated using the mean of all copy number segments overlapping the gene coordinates, weighted by the proportion of the segment covering the total transcript length. Copy number loss, gain, and biallelic loss status were determined by incorporating tumor purity, tumor ploidy, autosomes, and sex chromosomes variables according to the following thresholds. Copy number < 0.5 was called biallelic loss. For single-copy sex chromosomes, copy gain was called if the weighted mean copy number > (tumor ploidy × 0.8), and copy loss was called if the weighted mean copy number < (tumor ploidy × 0.3). For autosomal chromosomes, copy gain was called if the weighted mean copy number > (tumor ploidy × 1.95), and copy loss was called if the weighted mean copy number was < (tumor ploidy × 0.5).

### Complex structural variant identification and classification

Complex Structural Variants (cSV) identifications were performed with AmpliconArchitect (AA) [37] following the author's recommended settings with modifications described below. First, fastq files were re-aligned against the AA curated repository genome reference GRCh38 using the docker image *PrepareAA* version 0.1203.1. Copy number calls generated as described above using PURPLE were smoothed using the *seed_trimmer.py* script from *PrepareAA* with default parameters (–minsize 50,000 and –cngain 4.5). The output bed file was provided to the *amplified_intervals.py* script from AmpliconArchitect version 1.2 with optimized parameters (–gain 5 –cnsize_min 100,000). The *AmpliconArchitect.py* script was executed with the seed interval list, mapped reads, and parameters (–ref GRCh38 –downsample −1 –extendmode EXPLORE –sensitivems False –plotstylesmall –insert_sdevs 3.0 –pair_support_min 2). Classification of the identified amplicon structures was performed by AmpliconClassifier [20] (AC) version 0.4.9 with the following parameters: (–ref GRCh38 and –plotstyle individual –min_flow 1 –min_size 5000 –decomposition_strictness 0.1). The similarity score and Jaccard Breakpoint Index were calculated using the AmpliconClassifier script *amplicon_similarity.py*. To represent the potential structures, the amplicon cycles file was uploaded to genomequery.ucsd.edu:8800. This allowed visualization and combination of the structures, such as combining the overlapping segments from cycle 1, and cycles 3 and 9 to represent larger cycles 15 and 16.

### Determining chromothripsis

Chromothripsis was evaluated as previously described [6] with the following modifications. In brief, we quantified the insertions and deletions detected by GRIDSS, and we used the copy number calls from PURPLE within a moving of 2.5 Mb window positioned at 10 Kb intervals. Chromothripsis was identified when we detected at least 15 inversions, 15 copy number switches, and 10 deletions.

### Transcriptome processing, clustering, and signature analysis

RNA-seq Fastq files were pseudo-aligned and quasi-mapped to GENCODE 28) using Kallisto [79] version 0.46.1 with the following parameters: ‘kallisto quant –bootstrap-samples 100 –pseudobam’. Transcript read counts and abundance were summarized to gene-level using R package tximport (http://bioconductor.org/packages/tximport). Mitochondrial, ribosomal RNA and ribosomal pseudogenes were excluded from the counts and Transcripts Per Million (TPM) was recalculated for downstream analysis. Supervised hierarchical clustering and cluster assignment of the five subtypes in [32] was performed using ConsensusClusterPlus [80] version 1.60.0 R package with the following parameters: maximum cluster number = 6, number of subsamples = 50, item resampling = 0.8, gene resampling = 1, cluster algorithm = pam, distance = euclidean. We averaged log2(TPM + 1) and converted TPM values to z-scores for cross-sample clustering. To ensure robust discrimination between the subtypes, transcriptional signatures scores were calculated using GSVA [81] version 1.44.5 R package. Specifically, androgen pathway activity was calculated based on the z-scores of 21 genes reported to be induced by androgen [82] and NEPC score was calculated using 27 genes up-regulated in NEPC [28].

### Hi-C libraries and data processing

Most WGS samples (*n* = 80) were previously sequenced and are available at the European Genome-phenome Archive (EGA) (accession no. EGAS00001006604). One additional sample was sequenced using the same DNA extraction and library preparation methods as for the published samples [27]. Briefly, paired-end raw reads of the Hi-C libraries were processed using HiC-Pro [83] v.3.0.0. Reads were aligned to the reference human genome (hg38) using Bowtie2 [84] with the ‘-very-sensitive’ option. To rescue the unmapped chimeric fragments spanning the ligation junction, the ligation site was detected using an exact matching procedure; the 5′ fraction of the reads were aligned back to the reference genome. Unmapped reads, multimapped reads, and singletons were then discarded. Each pair of aligned reads was then assigned to MboI restriction fragments. Read pairs from uncut DNA, self-circle ligation, and PCR artifacts were filtered out, and the valid read pairs involving two different restriction fragments were used to build the contact matrix. Valid read pairs were then binned at a 10 Kb resolution. The binned contact matrix was then normalized using the iterative correction method [85] to correct for biases such as GC content, mappability, and effective fragment length in the Hi-C data.

To complement the WGS-based analysis of cSVs, we evaluated their impact on 3D genome structure using Hi-C features associated with copy number changes, structural variants, and ecDNA. We first stratified cSVs into four categories based on their structural complexity: (1) cSVs involving a single segment from one chromosome; (2) cSVs involving multiple segments from one chromosome; (3) cSVs involving one or multiple segments from two chromosomes; and (4) cSVs involving one or multiple segments from more than two chromosomes.

For categories (1) and (2) (Fig. 2b), we designed a statistical framework to assess local differences in cis-interactions. Specifically, we compared Hi-C contact frequencies within cSV-associated segments to two unamplified neighboring regions of equal length upstream and downstream of the cSV (referred to as the flanking region). In addition, we compared the contact frequencies of cSV-associated segments (cSV projection) to those of the upstream and downstream unamplified flanking regions (flank projection).

For categories (3) and (4) (Fig. 2d), we extended this framework to assess trans-interactions. First, we calculated the intra-cSV contact frequencies across all segments belonging to the same cSV. Next, for each chromosome contributing to the cSV, we compared contact frequencies within cSV-associated segments of one chromosome to the flanking regions of the partner chromosome(s) (cSV-overlapping). We also evaluated differences in contact frequencies within upstream and downstream unamplified regions on each chromosome (non-overlapping).

Statistical differences in contact frequencies were evaluated using the Wilcoxon rank-sum test. For cSVs spanning multiple segments and/or chromosomes, *p*-values were adjusted for multiple testing using the Benjamini–Hochberg (BH) procedure. For categories (1) and (2), we considered Hi-C data to provide orthogonal evidence when the comparison between cSV projection and flank projection showed at least one segment with a statistically significant difference after BH correction (adjusted *P* < 0.05). For categories (3) and (4), orthogonal evidence was defined as at least one significant comparison after BH correction (adjusted *P* < 0.05) between cSV-associated segments and the non-overlapping flanking regions.

For a subset of Hi-C samples, we performed loop calling following the approach described by Dehkordi and colleagues [21] to assess whether the fold-back inversions characteristic of BFB events generated distinctive Hi-C contact patterns. HiC-Pro formatted data were converted to the cool format, and multi-resolution cool files (1 kb, 10 kb, and 40 kb) were generated using the hicConvertFormat tool from the HiCExplorer version 3.7.6 suite of tools [86, 87]. Copy number was calculated to remove copy number variation effects from Hi-C data using the calculate-cnv and correct-cnv modules in NeoLoopFinder version 0.4.3 [88] at all three resolutions. Structural variations were predicted using the CNV-normalized matrices calculated by NeoLoopFinder as input for the predictSV tool and reformatted using the reformatSV tool from EagleC2 version 2.1.1 [89]. Assembly of complex SVs was performed using the assemble-complexSVs module in NeoLoopFinder, with the list of reformatted SVs and the CNV-corrected Hi-C matrix for the same samples as inputs. Identification of neo-loops across SV breakpoints was performed by manually adding the SV breakpoint collection derived from AA to the assembly files generated in the previous step and using them as input to the neoloop-caller module in NeoLoopFinder. Hi-C contact maps were plotted using *GENOVA* [90]*,* version 1.0.1, and genomic tracks were visualized using *gGnome version 0.1* [91]*.*

### Identification of regulatory regions amplified in cSVs

To characterize regulatory regions associated with cSVs, we first intersected cSV coordinates with H3K27ac consensus peaks generated from primary prostate tumors and patient-derived xenografts, as reported by Pomerantz et al [92]. Consensus peaks and cSV intervals were imported as GRanges objects, and overlaps were identified using the join_overlap_inner function from the plyranges package in R with a minimum overlap of 100 bp (minoverlap = 100). cSVs were considered positive if they intersected at least one H3K27ac peak under these criteria. In a complementary analysis, we annotated super-enhancer regions using the dbSUPER database [51]. cSVs were defined as overlapping a super-enhancer if any portion of the ecDNA or BFB interval intersected with a dbSUPER region. All genomic intervals were processed using the hg38 reference build to ensure consistency across datasets.

### Permutation test for cSV enrichment with enhancer sites

The significance of the overlap between ecDNA or BFB segments and H3K27ac peaks was assessed using the regioneR R package [93]. We applied the overlapPermTest function with 10,000 permutations, randomizing input intervals while preserving their size and chromosome distribution, and masking unmappable regions using the ENCODE list of excluded regions [94] (ENCFF356LFX). Empirical *P* values were derived from the null distribution of overlaps.

### Statistical analysis

Statistical analysis was performed in the R environment version 4.2.0 [95]. Mann–Whitney and Pearson’s Chi-squared tests were performed and visualized using *ggstatsplot* version 0.9.5. Other data visualization was performed using *ComplexHeatmap* version 2.12.1, *ggplot2* version 3.4.1, and *Gviz* version 1.41.2.

## Supplementary Information

Additional file 1: Figures S1 through S8.

Additional file 2: Tables S1 through S15.

## Data Availability

Code to reproduce figures and analysis is available at https://github.com/DavidQuigley/CASCADE_MCRPC [96], released under the MIT open source license. Raw data from the WCDT WGS, RNA, and Hi-C sequencing samples are available at dbGAP (accession number phs001648 [97]) and the European Genome-Phenome Archive (EGA) (accession numbers EGAS00001006649 [98], EGAD00001008487 [99], EGAS00001006604 [100]). The deep WGS from ctDNA from prostate cancer patients has been previously published^11^ under the accession code EGAS00001005783 [101] and is available under standard EGA controlled release. Raw data from the CASCADE cohort are available at EGA (accession EGAS00001007147 [102]). Additional data to reproduce analysis in this manuscript was deposited in Zenodo (zenodo.org) at 10.5281/zenodo.8347487 [103].
