# Supplementary material for: Convergent evolution of complex structural variants drives therapy resistance in metastatic prostate cancer
Source: Genome Biol. 2026 Apr 15;27:177. doi: 10.1186/s13059-026-04074-2 (PMC13191960; doi:10.1186/s13059-026-04074-2)
Supplement: Supplementary file 1 — Additional file 1: Figures S1 through S8. [file 13059_2026_4074_MOESM1_ESM.pdf]

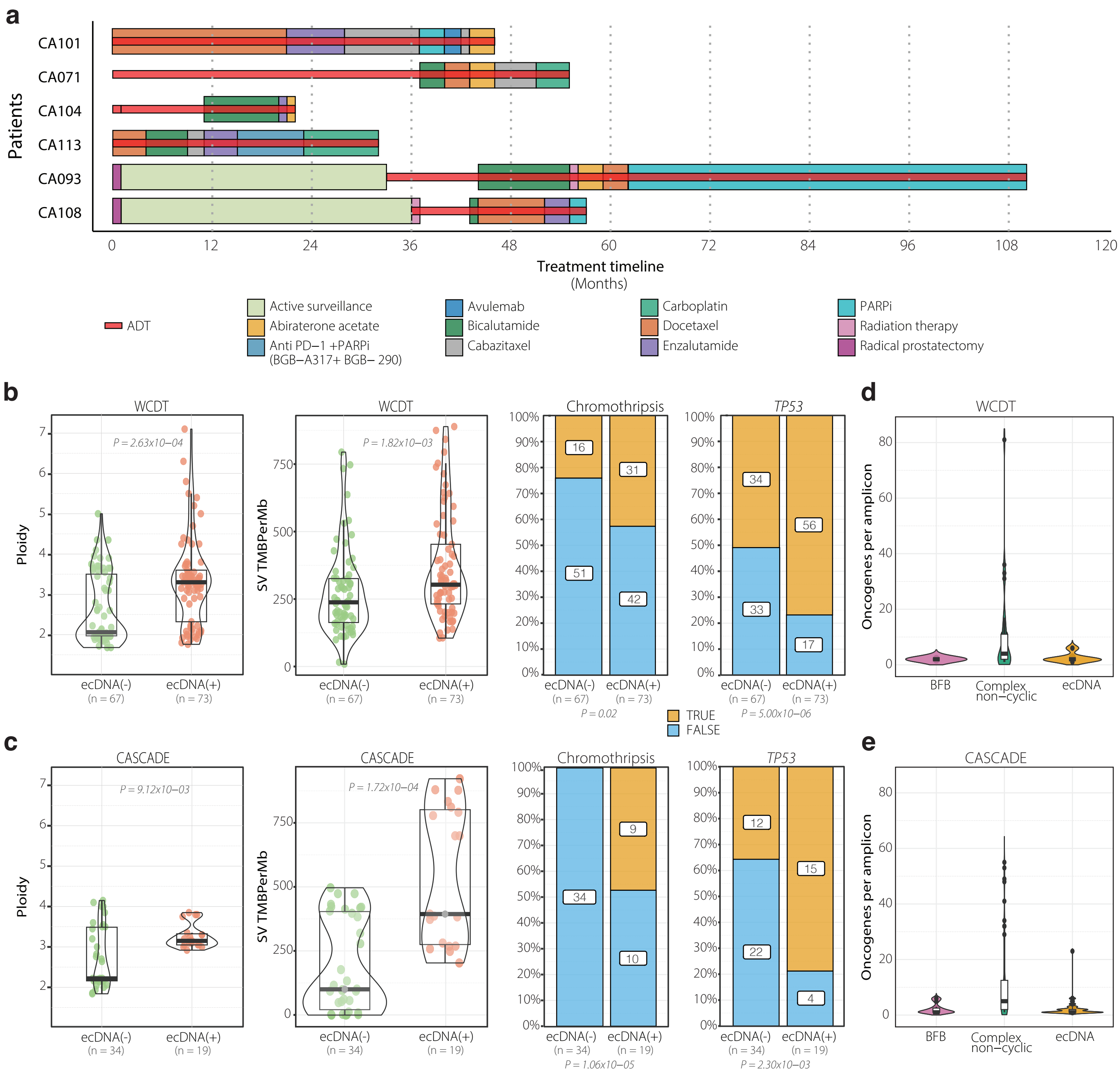


Fig. S1. CASCADE patients' therapy history and correlates of ecDNA with genome instability, chromothripsis, and *TP53* alterations. (a) Horizontal bar plots summarizing treatment history of CASCADE patients. All patients were on ADT (red bars); additional treatments and treatment combinations are color-coded in the legend. **(b-c)** Statistically significant associations between the presence of ecDNA and genomic alterations in WCDT and CASCADE cohorts. Box plots show the presence of ecDNA is associated with higher ploidy and higher structural variant burden per Mb (SV tmbPerMb) (Wilcoxon rank sum test) . Bar plots show ecDNA is associated with chromothripsis, and with alterations affecting *TP53* gene (Pearson's Chi-squared test) . **(d-e)** Number of oncogenes per amplicon in WCDT and CASCADE cohorts.

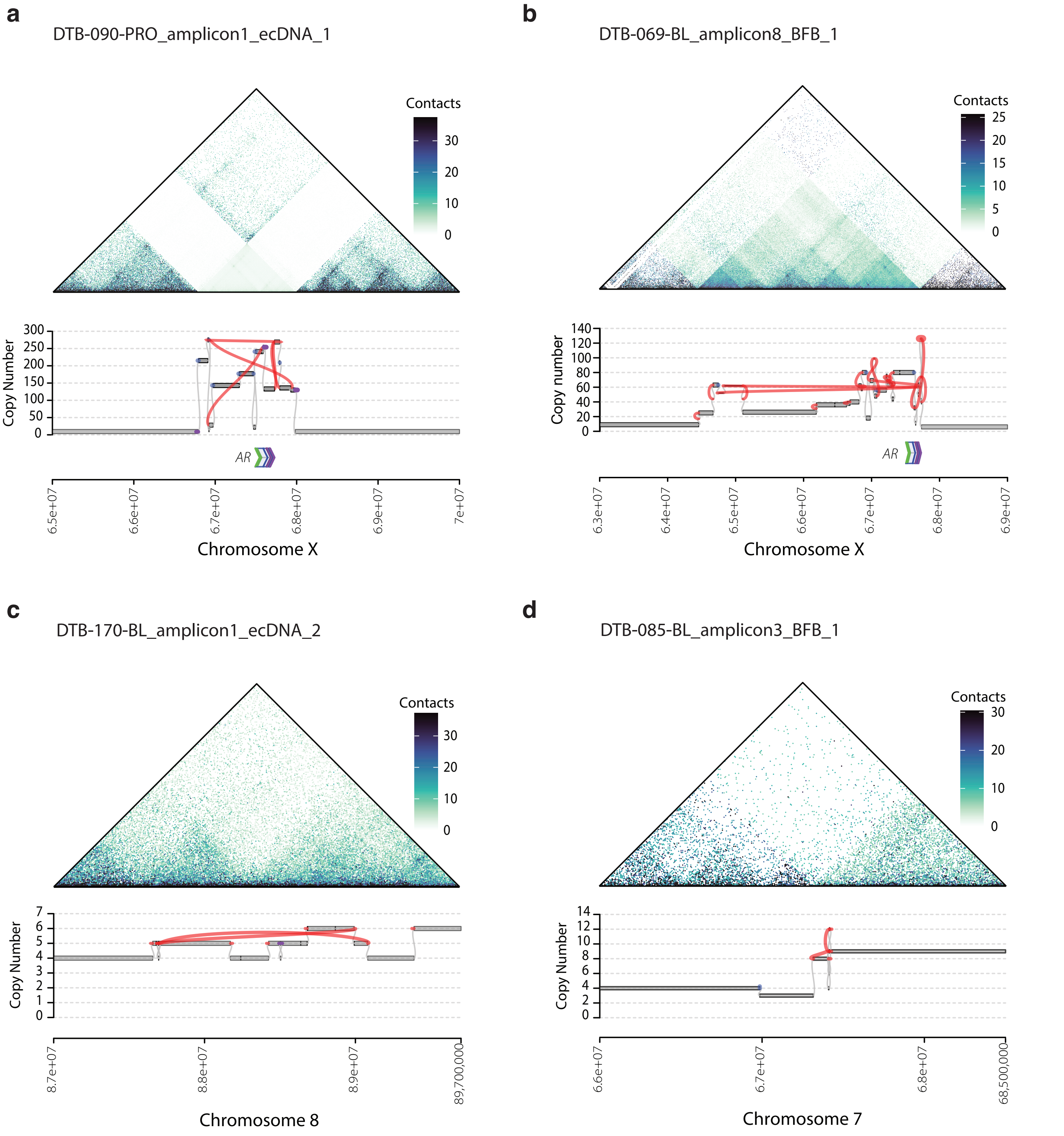


**Fig. S2. Concordance between AmpliconArchitect / AmpliconClassifier calls and Hi-C data. (a,b)** An ecDNA and BFB call supported by Hi-C analysis of matched tumors. (c) An ecDNA that was not supported by Hi-C analysis of the matched tumor. **(d)** A BFB call where the overall event was supported, but an individual segment was not supported.

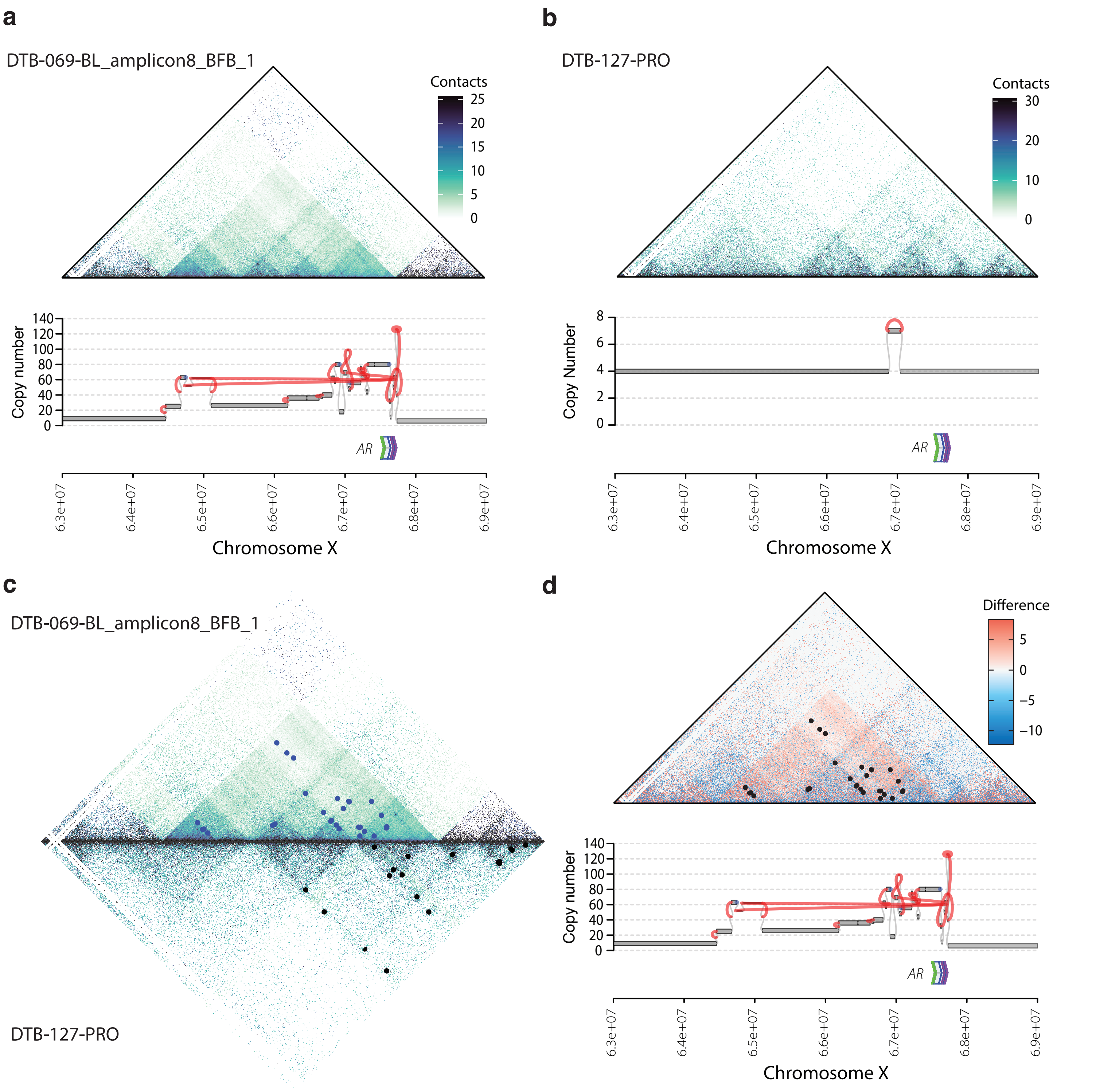


**Fig. S3. Hi-C contact maps and neo-loop annotation in mCRPC samples. (a)** Hi-C contact map (10 kb resolution) at the *AR* locus on chromosome X in an mCRPC tumor harboring a BFB amplicon at that locus, with reconstructed copy number segments shown below, connected via a genome graph representation of the BFB event. **(b)** Hi-C contact map (10 kb resolution) at the *AR* locus on chromosome X in an mCRPC tumor with no amplicons detected at that locus, with reconstructed copy number segments shown below. **(c)** Hi-C contact maps (10 kb resolution) across the subset of samples analyzed, with annotated neo-loops at BFB-predicted junctions marked by blue and black dots. **(d)** Hi-C contact map (10 kb resolution) showing the difference in contact frequency between two representative samples.

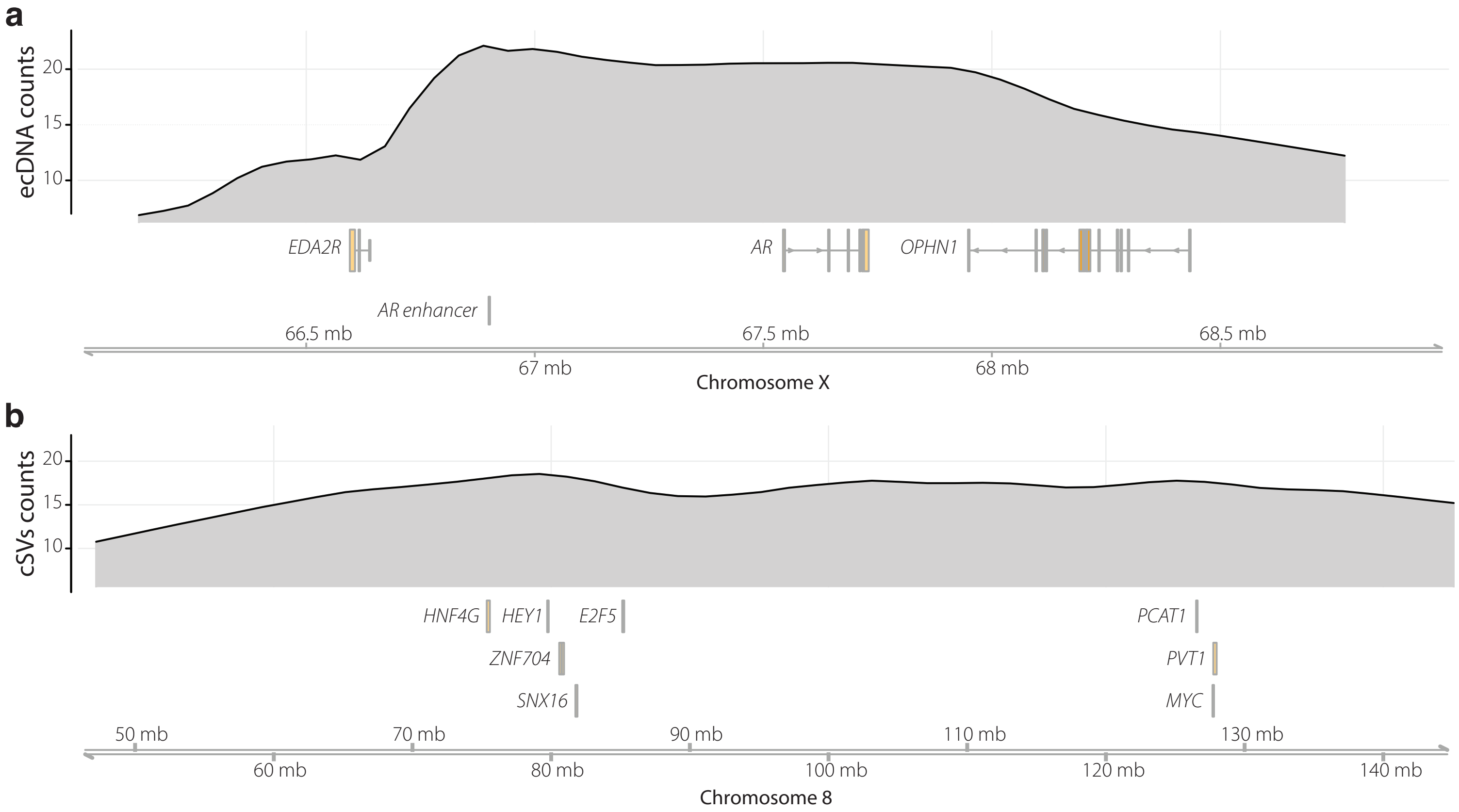


**Fig. S4. Distribution of cSVs targeting non-coding regulators in WCDT patients in three loci. (a)** Plot showing the frequency of ecDNA segments at the AR locus, with a peak at the AR enhancer previously identified[4]. **(b)** Plot showing the frequency of cSVs at the long arm of chromosome 8.

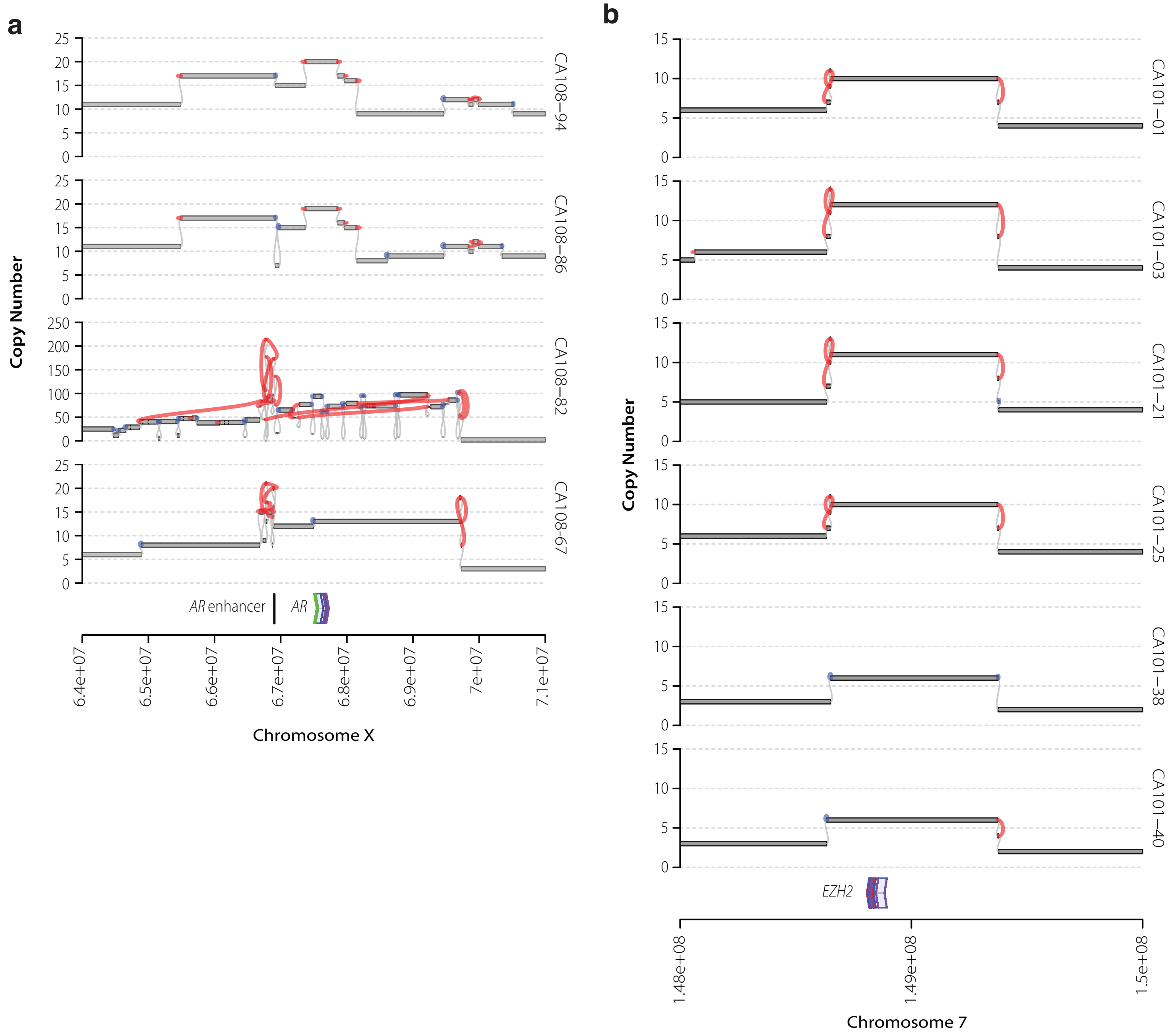


**Fig. S5. CASCADE patient CA108 and CA101 amplicons. (a)** Reconstructed genome graphs of distinct cSVs affecting the *AR* locus detected in patient CA108. From top to bottom, two ecDNAs, a BFB, and a linear amplification. **(b)** Genome graphs of the amplicons affecting the *EZH2* locus in patient CA101.

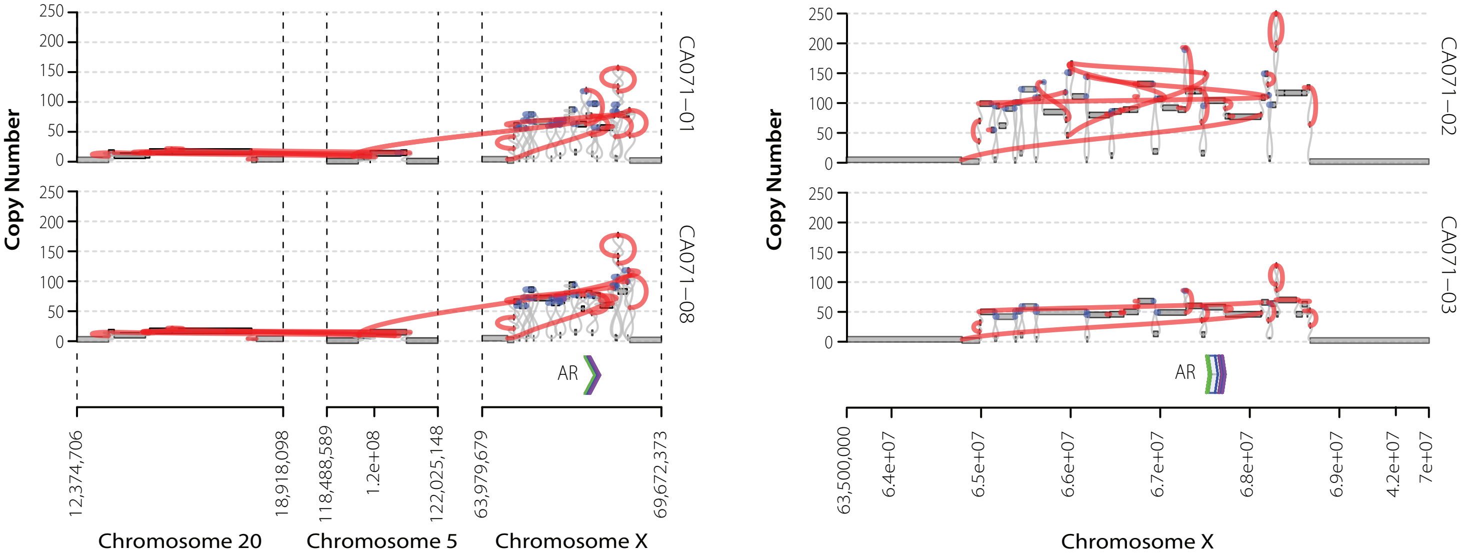


**Fig. S6. CA071 patient amplicons.** Samples CA071-01 and -08 harbored lower-level copy gain including chromosomes 5 and 20, while samples CA071-02 and -03 lacked these alterations.

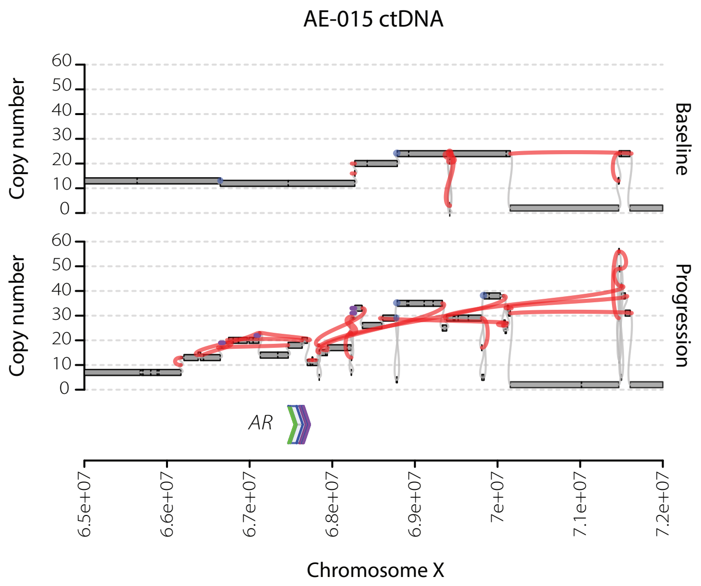


**Fig. S7. Patient gain in cSV complexity after progression on ARPI therapy.** In patient AE-015, this region of *AR* was identified as a linear amplification at baseline but an ecDNA at the time of progression on AR-directed therapy.

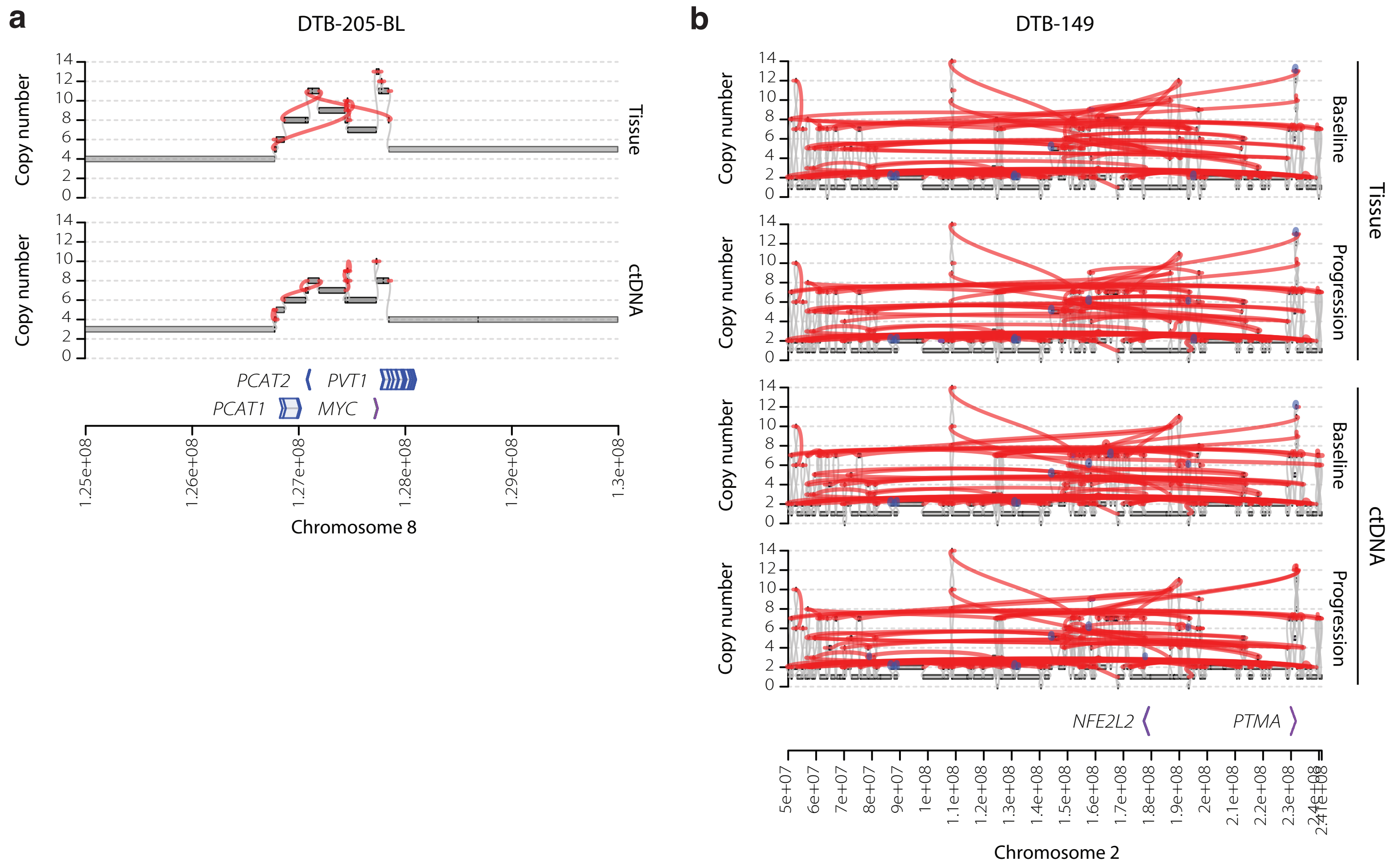


**Fig. S8. Additional examples of matched tumor and ctDNA ecDNA. (a)** Matched tissue and ctDNA samples from patient sample DTB-205 at baseline (top, sample DTB-205-BL) and contemporaneous ctDNA (bottom) illustrating an ecDNA amplifying the *MYC* locus on chromosome 8. **(b)** Tissue biopsies from patient DTB-149 obtained at baseline (first row) and after progression on AR-directed therapy (second row) demonstrating a complex ecDNA on chromosome 2. Contemporaneous ctDNA samples from patient DTB-149 at the time of baseline biopsy (third row) and progression biopsy (fourth row) demonstrate additional cases where ecDNA was identified in tissue biopsy and contemporaneously sampled ctDNA.
